# Histology-driven tailoring of surgical approaches in retroperitoneal soft tissue sarcoma: retrospective cohort study

**DOI:** 10.1093/bjsopen/zraf050

**Published:** 2025-05-13

**Authors:** Julian Musa, Franziska Willis, Ingmar F Rompen, Julian-Camill Harnoss, Thomas G P Grünewald, Mohammed Al-Saeedi, Markus W Büchler, Martin Schneider

**Affiliations:** Department of General, Visceral, and Transplantation Surgery, University Hospital Heidelberg, Heidelberg, Germany; Division of Translational Paediatric Sarcoma Research (B410), German Cancer Research Centre (DKFZ), Heidelberg, Germany; Hopp Children’s Cancer Centre (KiTZ), Heidelberg, Germany; Department of General, Visceral, and Transplantation Surgery, University Hospital Heidelberg, Heidelberg, Germany; Department of General, Visceral, and Transplantation Surgery, University Hospital Heidelberg, Heidelberg, Germany; Department of General, Visceral, and Transplantation Surgery, University Hospital Heidelberg, Heidelberg, Germany; Division of Translational Paediatric Sarcoma Research (B410), German Cancer Research Centre (DKFZ), Heidelberg, Germany; Hopp Children’s Cancer Centre (KiTZ), Heidelberg, Germany; Institute of Pathology, Heidelberg University Hospital, Heidelberg, Germany; Department of General, Visceral, and Transplantation Surgery, University Hospital Heidelberg, Heidelberg, Germany; Department of General, Visceral, and Transplantation Surgery, University Hospital Heidelberg, Heidelberg, Germany; Department of General, Visceral, and Transplantation Surgery, University Hospital Heidelberg, Heidelberg, Germany

## Abstract

**Background:**

Histology-driven tailoring of surgical approaches for retroperitoneal soft tissue sarcoma is currently under debate. Compelling evidence assessing the role of histology-dependent extent of resection is lacking. The aim of this study was to assess outcomes of patients with primary retroperitoneal liposarcoma (LPS) or leiomyosarcoma (LMS) according to whether comprehensive (formerly ‘compartmental’) resection (CR) was performed.

**Methods:**

A retrospective study was conducted on data from patients undergoing surgical resection for LPS and LMS at Heidelberg University Hospital (2002–2019). Parameters were compared between groups with and without CR, with subgroup analyses for grading (LPS). Kaplan–Meier and Cox regression analyses were used to identify predictors of disease-specific survival (DSS), local recurrence-free survival, and distant metastasis-free survival.

**Results:**

In total, 119 patients with primary LPS and 46 patients with primary LMS were identified. DSS was improved in patients with LPS with CR (*P* = 0.049), and both DSS (*P* = 0.040) and distant metastasis-free survival (*P* = 0.041) were improved in the subgroup of patients with primary G3 LPS. In contrast, CR in patients with LMS was not associated with improved DSS, local recurrence-free survival, or distant metastasis-free survival. CR was associated with more severe postoperative complications (*P* = 0.021) and a longer hospital stay (*P* = 0.013) in patients with LPS, longer operation times (*P* < 0.010) in both patients with LPS and LMS, and increased blood loss (*P* = 0.008) in patients with LMS.

**Conclusion:**

CR is associated with improved DSS in patients with primary LPS, which is not the case in patients with primary LMS. Given the association between CR and increased perioperative morbidity, surgical strategies for retroperitoneal soft tissue sarcoma should be individualized according to the underlying histology.

## Teaser

This study evaluated the outcomes of comprehensive resection (CR) in patients with primary retroperitoneal liposarcoma (LPS) and leiomyosarcoma (LMS). CR was associated with improved disease-specific survival in LPS patients, but not in LMS patients. Despite its advantages in LPS, CR was associated with higher postoperative morbidity, suggesting that surgical approaches for retroperitoneal soft tissue sarcomas should be tailored according to histological subtype.

## Introduction

Retroperitoneal soft tissue sarcomas (RPS) comprise a histologically heterogeneous group of malignant tumours that primarily affect patients around their sixth decade of life, with an annual incidence rate of 0.76 cases per 100 000 individuals^1–3^. Retroperitoneal liposarcoma (LPS) is the most frequent (45–55%), comprising well differentiated LPS (WDLPS, G1; 15–24%) and dedifferentiated LPS (DDLPS, G2/3; 16–21%), followed by retroperitoneal leiomyosarcoma (LMS; 21–26%)^2,4^. RPS with different histology exhibit distinct clinical phenotypes, creating specific clinical challenges that account for tumour-related mortality^5^. Local recurrence frequently occurs in both WDLPS and DDLPS, whereas additional metastatic spread is often seen in G3 DDLPS, which worsens prognosis^5,6^. In LMS, patient prognosis is predominantly determined by distant metastases, with local recurrence being less frequent than in LPS^5,6^.

The mainstay of treatment for RPS is gross macroscopic tumour resection including adjacent organs^7–12^. Modifying surgical approaches according to tumour type, grading, and clinical features (tumour biology) is increasingly being recommended^6,13^. Retroperitoneal WDLPS and G2 DDLPS predominantly exhibit a tendency for local recurrence, with WDLPS posing virtually no risk of metastatic spread and G2 DDLPS presenting only a low risk of distant metastases^6,13^. Differentiating WDLPS from normal retroperitoneal adipose tissue can be challenging, whereas G2 DDLPS are generally more readily distinguishable^6,13^. However, due to intratumour heterogeneity, marginal G1 components in G2 and G3 DDLPS are also difficult to distinguish from normal retroperitoneal adipose tissue. Therefore, comprehensive (formerly ‘compartmental’) resection (CR) has been proposed as an extended surgical strategy to enhance the likelihood of achieving negative surgical margins^6,13^. CR was originally defined as the systematic resection of uninvolved contiguous organs surrounding the tumour to ensure free surgical margins, and typically comprises *en bloc* resection of the hemicolon, kidney, and part of the psoas, as well as additional adjacent organs as necessary^14^. Although metastatic spread is more likely to determine oncological outcomes in retroperitoneal G3 DDLPS than in WDLPS or G2 DDLPS, local recurrence remains a significant concern, with a 5-year local recurrence rate of 33% and a distant metastasis rate of 44%^15^. Therefore, CR is still considered appropriate for these tumours, because local recurrence remains a predominant form of disease recurrence, necessitating aggressive surgical strategies to maximize local control^13^. In contrast, in LMS, tumour margins tend to be clearly distinguishable from surrounding tissues, which is why resection of adherent structures while leaving the remainder of the compartment intact has been discussed as an appropriate surgical treatment^6,13^. However, current recommendations concerning histology-driven surgical approaches rely on a few retrospective studies, which did not systematically assess the role of CR as an independent prognostic factor for oncological outcome depending on tumour histology^14,16,17^.

The primary aim of the present study was to investigate the impact of CR based on tumour histology, as well as tumour biology and clinical presentation. This approach aims to provide a more comprehensive understanding of the significance of CR in the treatment of RPS to potentially refine surgical strategies to improve patient outcomes.

## Methods

Following approval by the Ethics Board of the University of Heidelberg (S-649/2012), a retrospective analysis was performed of all patients who underwent resection of primary retroperitoneal LPS and LMS between January 2002 and December 2019 at the Department of General, Visceral, and Transplantation Surgery, Heidelberg University Hospital. Patients were analysed based on clinicopathological parameters and clinical outcomes with a particular emphasis on the conduction of CR.

The variables assessed were age at diagnosis, sex, grading according to Fédération Nationale des Centres de Lutte Contre le Cancer (FNCLCC), tumour size, number of organs resected, conduction of CR, resection status (R0/R1 or R2), postoperative complications (as assessed by Clavien–Dindo classification and the comprehensive complication index [CCI]^18^), intraoperative blood loss, requirement for blood transfusions, operation time, duration of hospital stay, reoperations, (re-)interventions (defined as non-surgical, but invasive, procedures, such as computed tomography-guided drainage placement), 30-day and 90-day mortality, the application of radiotherapy or systemic therapy, disease-specific survival (DSS), local recurrence-free survival (LRFS), distant metastasis-free survival (DMFS), the crude cumulative incidence of local recurrences, and the crude cumulative incidence of distant metastases.

Microscopic surgical margins were assessed in a reference centre by senior pathologists experienced in retroperitoneal sarcomas on conventional haematoxylin- and eosin-stained slides; in case of doubt, additional analyses were performed, including chromogen *in situ* hybridization for the *MDM2* gene and/or immunohistochemical staining for MDM2 and/or cyclin dependent kinase 4 (CDK4). With regard to tumour grading, the term ‘not applicable’ was used for patients who had undergone neoadjuvant treatment because, following such treatment, the pathology report does not generally include tumour grading, which is determined by the examining pathologist and the particular characteristics of the tumour.

DSS was defined as the interval between the date of surgery and the date of death due to RPS. LRFS and DMFS were defined as the interval between the date of surgery and the date of occurrence of a local recurrence or distant metastasis, respectively. Local recurrence was defined as tumour recurrence in the retroperitoneum or abdomen, excluding liver metastases. Liver metastases and all other metastases were defined as distant metastases. For surviving patients, follow-up was censored at the date of last disease assessment.

CR was defined as ipsilateral hemicolectomy, nephrectomy, and resection of the fascia or psoas muscle as a minimum, sometimes with additional adrenal resection, splenectomy, (partial) pancreatectomy, and resection of the inferior vena cava, iliac vessels, and other organs, if involved. The concept of CR was gradually implemented upon increasing evidence in the field, enabling retrospective investigation of a CR *versus* no CR group (*Table S1*). In the remaining 25–30% of patients who did not undergo CR in recent years, the extent of the resections was less due to individual patient factors, as determined at the discretion of the operating surgeon.

To identify significant differences between categorical and metric variables according to CR, χ^2^ and Mann–Whitney *U* tests were performed. Kaplan–Meier analyses with log-rank statistics were used for survival analyses. DSS, LRFS, and DMFS are presented as the median with 95% confidence interval (c.i.) after 5 years of follow-up, along with 3-year and 5-year survival rates. For analysis of the crude cumulative incidence of local recurrences and distant metastases, death was treated as a competing event and the Fine–Gray test was used to test for statistical significance^19^.

Multivariable Cox regression analyses were performed to identify independent prognostic factors for DSS, LRFS, and DMFS. Patient age was modelled as a continuous variable, all other variables were modelled as categorical variables using dummy variables. In respect of number of events, backwards step-wise variable selection was used^20^. For analyses of LRFS, patients with macroscopically incomplete resection (R2) were excluded. Analyses were performed for primary LPS and primary LMS separately. Due to different LPS recurrence patterns depending on tumour grade, subgroup analyses were performed for primary G2 LPS and G3 LPS. There was an insufficient number of patients with primary G1 LPS to enable subgroup analysis.

SPSS (Version 29.0.2.0) was used for all statistical analyses. The level of statistical significance was set to *P* < 0.05 (two-tailed).

## Results

### Patient characteristics and clinicopathological features

In total, 370 patients with RPS were operated at the University of Heidelberg between 2002 and 2019. Of these patients, 290 presented with LPS or LMS; 165 exhibited primary disease (119 primary LPS and 46 primary LMS) and were included in the present study.

The cohort comprises 119 patients with primary LPS (72.1%; 26 WDLPS (21.9%), 51 G2 DDLPS (42.9%), 29 G3 DDLPS (24.4%), and 13 without grading after neoadjuvant therapy (10.9%)) and 46 patients with LMS (27.9%; 18 G2 LMS (39.1%), 19 G3 LMS (41.3%), and 9 without grading after neoadjuvant therapy (19.6%)). The general clinicopathological characteristics of patients with primary LPS and LMS stratified according to the conduction of CR are presented in *Table 1*.

**Table 1:** Clinical and histological characteristics of the primary LPS and LMS cohorts according to whether CR was performed

	LPS			LMS		
	No CR	CR	P*	No CR	CR	P*
No. of patients	54	65		24	22	
Age at diagnosis (years), median (i.q.r.)	55.0 (50.5–66.0)	61.0 (53.5–72.0)	0.039	57.0 (51.5–68.8)	56.5 (49.3–68.3)	0.560
**Sex**			0.421†			0.553†
Male	31 (57.4%)	42 (64.6%)		9 (37.5%)	11 (50.0%)	
Female	23 (42.6%)	23 (35.4%)		15 (62.5%)	11 (50.0%)	
**Tumour grading**			0.257†			0.045†
G1	13 (24.1%)	13 (20.0%)				
G2	19 (35.2%)	32 (49.2%)		12 (50.0%)	6 (27.3%)	
G3	16 (29.6%)	13 (20.0%)		10 (41.7%)	9 (40.9%)	
Not applicable	6 (11.1%)	7 (10.8%)		2 (8.3%)	7 (31.8%)	
Tumour size (cm), median (i.q.r.)	21.8 (11.0–31.4)	20.0 (13.0–27.5)	0.519	9.5 (6.5–13.3)	11.5 (6.4–14.5)	0.488
No. of resected organs, median (i.q.r.)	2.0 (1.0–3.0)	4.0 (3.0–5.0)	<0.001†	2.0 (1.0–3.0)	3.5 (3.0–4.0)	<0.001†
**Resection status**			0.019†			0.509†
R0/R1	48 (88.9%)	65 (100.0%)		22 (91.7%)	22 (100.0%)	
R2	6 (11.1%)	0 (0.0%)		2 (8.3%)	0 (0.0%)	
**Systemic therapy**			0.112†			0.927†
No	50 (92.6%)	64 (98.5%)		22 (91.7%)	20 (90.9%)	
Before surgery	0 (0.0%)	0 (0.0%)		1 (4.2%)	1 (4.5%)	
After surgery	1 (1.9%)	1 (1.5%)		1 (4.2%)	1 (4.5%)	
Before and after surgery	3 (5.6%)	0 (0.0%)		0 (0.0%)	0 (0.0%)	
**Radiation therapy**			0.113†			0.322†
No	36 (66.7%)	34 (52.3%)		15 (62.5%)	5 (22.7%)	
Before surgery	7 (13.0%)	24 (36.9%)		7 (29.2%)	13 (59.1%)	
After surgery	11 (20.4%)	6 (9.2%)		2 (8.3%)	4 (18.2%)	
Before and after surgery	0 (0.0%)	1 (1.5%)		0 (0.0%)	0 (0.0%)	
Complications (Clavien–Dindo ≥III)	7 (13.0%)	20 (30.8%)	0.021†	6 (25.0%)	6 (27.3%)	0.861†
CCI score, median (i.q.r.)	26.2 (8.7–37.2)	29.6 (12.2–37.1)	0.466	26.2 (8.6–100.0)	42.6 (21.8–55.2)	0.971
(Re-)intervention	2 (3.7%)	10 (15.4%)	0.035†	2 (8.3%)	3 (13.6%)	0.564†
Reoperation	5 (9.3%)	9 (13.8%)	0.439†	2 (8.3%)	5 (22.7%)	0.175†
(Re-)intervention and Reoperation	0 (0.0%)	1 (1.5%)	0.360†	0 (0.0%)	0 (0.0%)	
30-day mortality	1 (1.9%)	3 (4.6%)	0.405†	4 (16.7%)	0 (0.0%)	0.045†
90-day mortality	3 (5.%)	3 (4.6%)	0.815†	4 (16.7%)	1 (4.5%)	0.187†
Operation time (min), median (i.q.r.)	225.0 (133.8–225)	270.0 (205.0–349.0)	0.006	225.0 (167.5–309.5)	352.5 (282.0–514.3)	0.002
Blood loss (ml), median (i.q.r.)	500.0 (300.0–500.0)	800.0 (325.0–1500.0)	0.149	700.0 (300.0–1650.0)	1475.0 (925.0–2700.0)	0.008
Transfusion	15 (27.8%)	19 (29.2%)	0.861†	10 (41.7%)	15 (68.2%)	0.085†
Length of hospital stay (days), median (i.q.r.)	10.0 (8.75–16.25)	21.8 (11.0–31.4)	0.013	15.0 (10.0–19.5)	15.50 (12.0–26.5)	0.197†
Follow-up duration (months), median (i.q.r.)	70.5 (21.7–121.7)	49.2 (24.7–92.5)	0.256	34.4 (12.9–89.9)	26.8 (16.2–63.3)	0.838
Follow-up duration if alive (months), median (i.q.r.)	87.3 (69.9–144.0)	63.6 (35.8–99.1)	0.005	97.7 (32.6–127.1)	52.2 (23.7–68.2)	0.142

(Re-)intervention refers to non-surgical, but invasive, procedures, such as computed tomography-guided drainage placement. *Mann–Whitney *U* test, except † χ^2^ test. LPS, retroperitoneal liposarcoma; LMS, retroperitoneal leiomyosarcoma; CR, comprehensive resection; i.q.r., interquartile range; CCI, comprehensive complication index (patients with complications only).

Of the patients with primary LPS, those who underwent CR were significantly older at diagnosis (*P* = 0.039) and had a greater median number of resected organs (*P* < 0.001) as well as a greater number of macroscopically complete tumour resections (*P* = 0.019) compared with those who did not undergo CR (*Table 1*).

Of the patients with primary LMS, those who underwent CR had fewer G2 tumours and a greater number of patients in whom tumour grading was not applicable (*P* = 0.045), likely due to higher rates of preoperative radiation, compared with patients who did not undergo CR (*Table 1*). Patients who underwent CR had an increased median number of resected organs (*P* < 0.001) (*Table 1*).

### Postoperative morbidity and mortality associated with CR

Of the patients with primary LPS, those who underwent CR experienced more severe postoperative complications (Clavien–Dindo ≥III; *P* = 0.021), accordingly requiring significantly more (re-)interventions (*P* = 0.035), and had longer durations of hospital stay (*P* = 0.013) compared with those who did not undergo CR (*Table 1*). However, when complications occurred, the severity as assessed by the CCI, did not differ significantly between the two treatment groups (*Table 1*). In addition, patients who underwent CR had longer operation times (*P* = 0.006) (*Table 1*).

Among patients with primary LMS, there were no significant differences in the occurrence of postoperative complications or in the length of hospital stay between those who did and did not undergo CR (*Table 1*). However, as for patients with primary LPS, operation times were longer (*P* = 0.002) and intraoperative blood loss was higher (*P* = 0.008) for patients with LMS who underwent CR (*Table 1*). Notably, among patients with primary LMS, 30-day mortality was significantly higher for those who did not undergo CR than for those who did undergo CR (*P* = 0.045) (*Table 1*).

### DSS, LRFS, and DMFS in patients with primary LPS

**Fig. 1:**
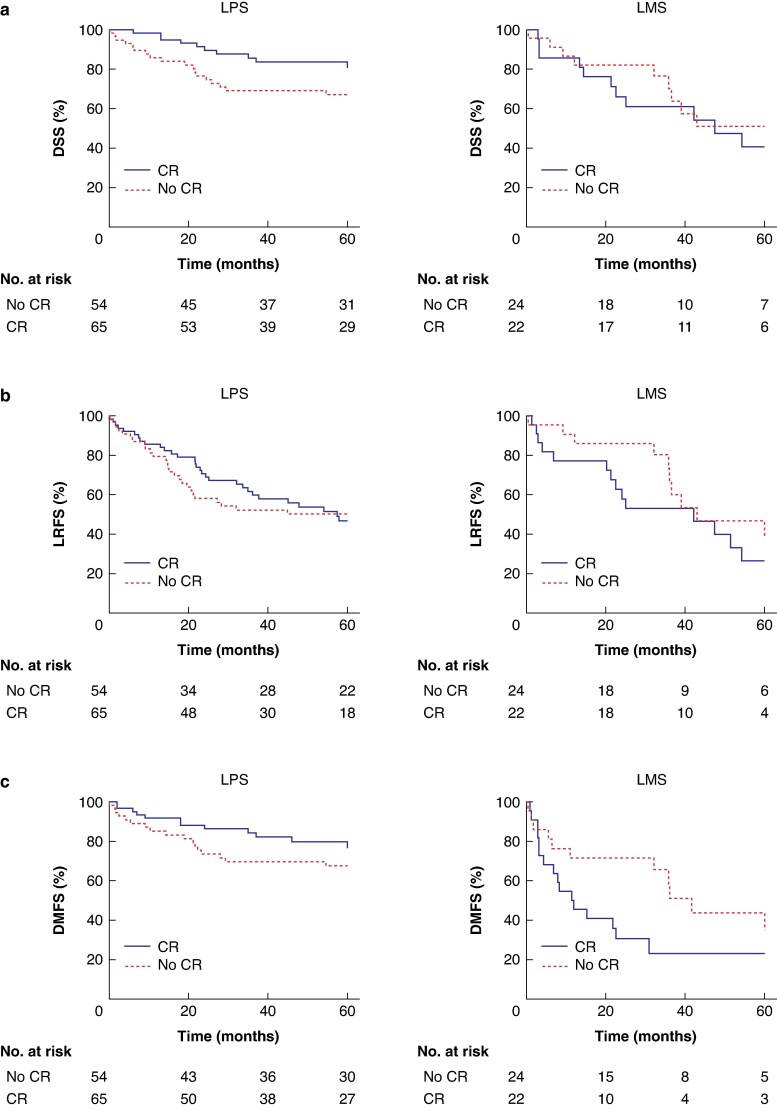
Kaplan–Meier curves for 5-year DSS, LRFS, and DMFS in patients with LPS or LMS, with or without CR **a** DSS, **b** LRFS, and **c** DMFS in primary LPS (left-hand panels) or LMS (right-hand panels). Significance levels between the CR and no CR groups were determined by log-rank tests and multivariable Cox regression. **a**  *P* = 0.058 (log-rank test) and *P* = 0.049 (multvariable Cox regression) for LPS; no significant difference (log-rank test and multivariable Cox regression) for LMS. **b** No significant difference (log-rank test and multivariable Cox regression) for LPS and LMS. **c** No significant difference (log-rank test and multivariable Cox regression) for LPS; *P* = 0.71 (log-rank test) and no significant difference (multivariable Cox regression) for LMS. LPS, retroperitoneal liposarcoma; LMS, retroperitoneal leiomyosarcoma; CR, comprehensive resection; DSS, disease-specific survival; LRFS, local recurrence-free survival; DMFS, distant metastasis-free survival.

Median DSS was not reached during the follow-up period, precluding its calculation. With CR, 3-year and 5-year DSS was 85 and 79%, respectively, compared with 70 and 67% without CR (*Fig. 1a*, *left panel*). In multivariable Cox regression analyses, CR was an independent prognostic factor and significantly associated with improved DSS (*P* = 0.049) independent of patient age, tumour grading, and resection status (*Table 2*). Similarly, younger age (*P* = 0.006) and G1 *versus* G3 grading (*P* = 0.023) were associated with improved DSS (*Table 2*).

**Table 2:** Multivariable Cox regression analysis for 5-year DSS, LRFS, and DMFS for patients with LPS and LMS

	LPS		LMS	
	HR	*P*	HR	*P*
**DSS**				
Age at first diagnosis	1.06 (1.02–1.1)	0.006		
Resection status (R0/R1 *versus* R2)			0.14 (0.01–1.39)	0.093
Comprehensive resection (yes *versus* no)	0.46 (0.21–1.00)	0.049	1.87 (0.73–4.83)	0.195
Tumour grade		0.074		0.254
G1 *versus* G3	0.09 (0.01–0.72)	0.023		
G2 *versus* G3	0.55 (0.24–1.27)	0.161	3.63 (0.70–18.85)	0.125
N/A *versus* G3	0.37 (0.10–1.40)	0.144	3.61 (0.76–17.13)	0.106
**LRFS**				
Age at first diagnosis	1.01 (0.99–1.04)	0.295	1.01 (0.98–1.04)	0.477
Comprehensive resection (yes *versus* no)	0.89 (0.50–1.59)	0.687	1.68 (0.73–3.89)	0.222
Tumour grade		0.002		
G1 *versus* G3	0.08 (0.02–0.36)	0.001		
G2 *versus* G3	0.87 (0.47–1.65)	0.678		
N/A *versus* G3	0.21 (0.05–0.95)	0.042		
**DMFS**				
Age at first diagnosis	1.05 (1.01–1.09)	0.012	0.98 (0.95–1.01)	0.163
Resection status (R0/R1 *versus* R2)				
Comprehensive resection (yes *versus* no)	0.58 (0.28–1.21)	0.148	2.21 (0.97–5.06)	0.060
Tumour grade		0.092		0.456
G1 *versus* G3	0.09 (0.01–0.70)	0.021		
G2 *versus* G3	0.69 (0.31–1.53)	0.362	1.80 (0.53–6.17)	0.348
N/A *versus* G3	0.41 (0.11–1.52)	0.182	2.08 (0.66–6.52)	0.210

Values in parentheses are 95% confidence intervals. LPS, retroperitoneal liposarcoma; LMS, retroperitoneal leiomyosarcoma; DSS, disease-specific survival; LRFS, local recurrence-free survival; DMFS, distant metastasis-free survival; HR, hazard ratio; N/A, not applicable.

Median LRFS was not reached during the follow-up period, precluding its calculation. With CR, 3-year and 5-year LRFS was 61 and 49%, respectively, compared with 57 and 55% without CR (*Fig. 1b*, *left panel*). Multivariable Cox regression analyses revealed that CR was not associated with improved LRFS (*Table 2*). Tumour grading was the only independent prognostic factor for LRFS in primary LPS (*P* = 0.002). The median interval to local recurrence was significantly longer in patients who underwent CR than in those who did not (25 (i.q.r.: 15.0–54.0) *versus* 15.5 (i.q.r.: 8.8–25.3) months, respectively; *P* = 0.023).

Median DMFS was not reached during the follow-up period, precluding its calculation. With CR, 3-year and 5-year DMFS was 83 and 75%, respectively, compared with 70 and 68% without CR (*Fig. 1c*, *left panel*). In multivariable Cox regression analyses, CR was not independently associated with improved DMFS (*Table 2*). Younger age (*P* = 0.012) and G1 *versus* G3 tumour grading (*P* = 0.021) were independently associated with improved DMFS (*Table 2*).

**Fig. 2:**
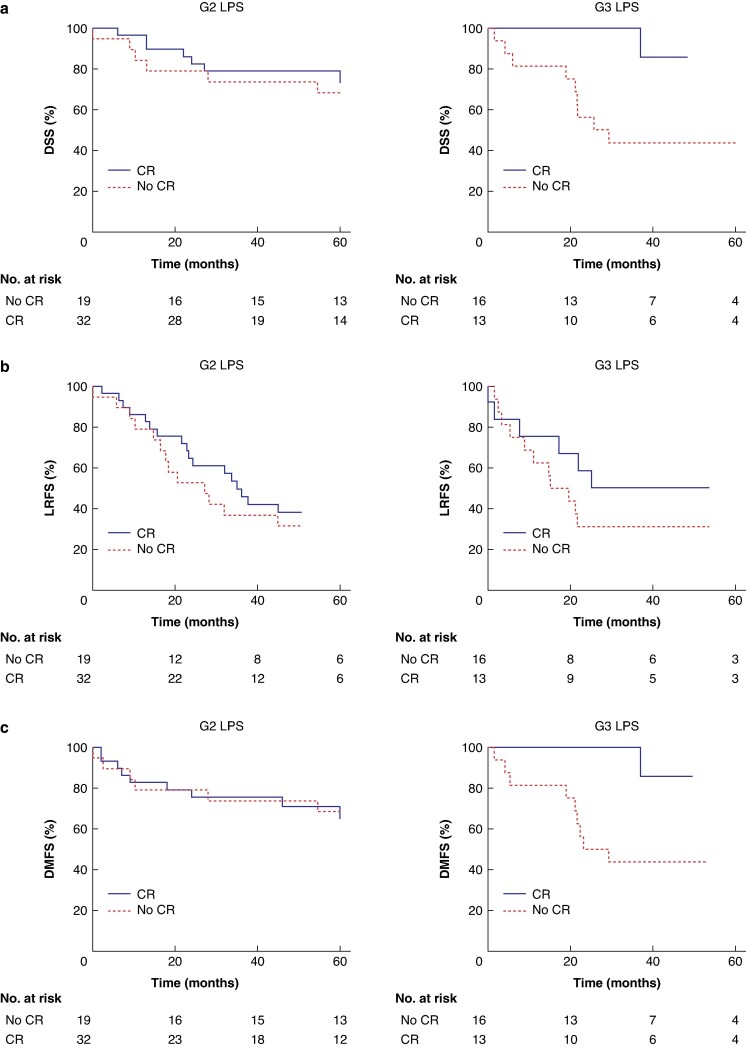
Kaplan–Meier curves for subgroup analyses of 5-year DSS, LRFS, and DMFS for primary G2 and G3 LPS, with or without CR **a** DSS, **b** LRFS, and **c** DMFS in primary G2 (left-hand panels) and G3 (right-hand panels) LPS. Significance levels between the CR and no CR groups were determined by log-rank tests and multivariable Cox regression. **a** No significant difference (log-rank test and multivariable Cox regression) for G2 LPS; *P* = 0.025 (log-rank test) and *P* = 0.040 (multivariable Cox regression) for G3 LPS. **b** No significant difference (log-rank test and multivariable Cox regression) for G2 and G3 LPS. **c** No significant difference (log-rank test and multivariable Cox regression) for G2 LPS; *P* = 0.027 (log-rank test) and *P* = 0.041 (multivariable Cox regression) for G3 LPS. LPS, retroperitoneal liposarcoma; LMS, retroperitoneal leiomyosarcoma; CR, comprehensive resection; DSS, disease-specific survival; LRFS, local recurrence-free survival; DMFS, distant metastasis-free survival.

When 5-year DSS, LRFS, and DMFS for primary G2 and G3 LPS were analysed separately, CR was found to be an independent prognostic factor for improved DSS in G3 tumours, but not in G2 tumours (*Fig. 2a*; *Table S2*); this was also true for DMFS (*Fig. 2c*; *Table S2*), but not LRFS (*Fig. 2b*; *Table S2*). Consistent with these findings, when depicting local recurrences and distant metastases as crude cumulative incidence rates with death as a competing event, a lower crude cumulative incidence of distant metastases in the CR compared with no CR group was found only for primary G3 LPS (*P* < 0.001) (*Fig. S1*).

CR has increasingly been implemented over the recent years. Kaplan–Maier and multivariable Cox regression analyses were performed separating patients according to the time period (TP1: 2002–2007; TP2: 2008–2013; TP3: 2014–2019) when the operation was performed. In these analyses, 5-year DSS increased significantly over time (log-rank *P* = 0.021) in LPS (*Fig. S2a*, *left panel*). Consistent with these findings, in LPS, the time period when the operation was performed showed a tendency to be an independent prognostic factor for DSS in multivariable analysis (*P* = 0.054), whereby comparison of TP1 and TP3 specifically was an independent prognostic factor for DSS (T1 *versus* T3: hazard ratio (HR) 3.51, *P* = 0.018) (*Table S3*). There were no significant differences in 5-year LRFS rates over the time in LPS (*Fig. S2b*, *left panel*) and, consistent with these findings, the time period when the operation was performed was not an independent prognostic factor in multivariable analyses (*Table S3*). However, 5-year DMFS increased significantly over the time in LPS (log-rank *P* = 0.039) (*Fig. S2c*, *left panel*). In multivariable analyses, the time period when the operation was performed reached borderline significance as an independent prognostic factor for DMFS in LPS (*P* = 0.050) and was statistically significant when comparing TP1 *versus* TP3 (HR 3.21, *P* = 0.025) and TP2 *versus* TP3 (HR 2.87, *P* = 0.029) separately (*Table S3*).

### DSS, LRFS, and DMFS in patients with primary LMS

Median DSS was not reached during the follow-up period, precluding its calculation. The 3-year and 5-year DSS with CR was 61 and 41%, respectively, compared with 70 and 51% without CR (*Fig. 1a*, *right panel*).

With CR, the median LRFS was 42.2 months (95% confidence interval (c.i.) 13.5 to 70.8 months), and the 3-year and 5-year LRFS was 53 and 27%, respectively (*Fig. 1b*, *right panel*). Without CR, the median LRFS was 42.9 months (95% c.i. 15.5 to 70.4), and the 3-year and 5-year LRFS without CR was 73 and 46%, respectively (*Fig. 1b*, *right panel*).

With CR, the median DMFS with CR was 11.4 months (95% c.i. 3.1 to 19.8 months), and the 3-year and 5-year DMFS with CR was 23 and 23%, respectively (*Fig. 1c*, *right panel*). Without CR, the median DMFS was 41.6 months (95% c.i. 31.9 to 51.3 months), and the 3-year and 5-year DMFS was 58 and 44%, respectively (*Fig. 1c*, *right panel*).

In multivariable Cox regression analysis, CR was not associated with improved DSS, LRFS or DMFS (*Table 2*). No independent prognostic factors for survival outcomes were identified (*Table 2*).

There was no significant association between the time point when the operation was performed and 5-year DSS, LRFS, and DMFS in univariate log rank and multivariable Cox regression analyses (*Fig. S2c*, *right panels*; *Table S3*).

## Discussion

This retrospective single-centre study of 165 patients with primary retroperitoneal LPS and LMS demonstrated that CR is an independent prognostic factor for improved DSS in patients with primary LPS. In patients with primary G3 LPS, CR seems to be linked to improved DMFS, which may contribute to improved DSS associated with CR. In contrast, such associations were not observed in LMS.

Given the distinct underlying tumour biology and corresponding clinical presentation of different types of RPS, histology-driven surgical approaches have recently been recommended^6,13^. However, these recommendations are based on a few retrospective studies that did not systematically assess the role of CR as an independent prognostic factor for oncological outcome depending on tumour type and histology^14,16,17^. These pioneering studies, which established the concept of CR, revealed that CR is associated with improved overall survival and LRFS in RPS compared with standard procedures such as simple complete excision of the tumour or *en bloc* resection of the tumour with tumour-infiltrated organs in grade 1 and 2 RPS^14,16,17^. However, these study cohorts were analysed according to specific time periods during which resections were performed (with CR being more frequent within specific periods), but not stratified according to the use of CR^16,17^. Furthermore, the cohorts were based on mixed RPS cohorts with different histologies^14,16,17^. When subgroup analyses were included, either grading across all RPS entities or RPS entities without considering tumour grade have been investigated separately^14,16,17^.

In contrast with previous reports that have shown an impact of CR on LRFS in RPS^14,16,17^, the present study did not detect a statistically significant difference regarding LRFS in primary LPS using either Kaplan–Meier or multivariable Cox regression analyses. Local recurrences were not reduced in the CR group, but the median time interval between initial tumour resection and the diagnosis of recurrence was significantly longer in patients with primary LPS who underwent CR. Indeed, a prolonged interval between diagnosis of tumour recurrence and the initial operation is recognized as an independent predictor of improved overall survival in primary RPS^9,21^, which may explain why CR was associated with improved DSS in the present cohort of patients with primary LPS.

Grading-stratified subgroup analyses of primary LPS performed in the present study revealed that CR appears to be associated with favourable DSS and DMFS, but not with LRFS in primary G3 LPS. In G3 LPS, the formation of distant metastases is a major determinant of prognosis^6^. These data suggest that upfront radical surgical treatment using CR may help prevent distant metastases, thereby contributing to improved DSS. However, these results and conclusions need to be interpreted with caution due to the small sample sizes in the respective subgroup analyses in this study. The lack of an association between CR and LRFS in G3 LPS further underscores the need for the validation of these findings in larger, preferably prospective, cohorts. In addition, these results should be contextualised within the ongoing STRASS-2 trial^22^, which is investigating the role of neoadjuvant chemotherapy in primary LMS and G3 LPS tumours.

In patients with primary LMS, there was no improvement in DSS, LRFS, or DMFS when CR was performed. However, the primary LMS cohort only included 46 patients in total, and the findings may not be generalizable to all patients with primary LMS. The present study demonstrates that patients with primary LMS do not appear to benefit from CR compared with *en bloc* resection of the tumour and visibly infiltrated organs. These findings support current recommendations^6,13^, which are based on LMS tumour biology and clearly distinguishable intraoperative tumour margins.

Personalization of surgical approaches regarding the extent of resection is of major clinical importance concerning surgical morbidity. The present study demonstrates that perioperative morbidity (intraoperative blood loss, number of severe complications, and length of hospital stay) is higher when performing CR. Although multivisceral resections in RPS are apparently not associated with inferior long-term quality of life^23^, increased morbidity rates associated with CR make it crucial to identify subgroups of patients with RPS that truly benefit in terms of oncological outcome. Given this importance of personalized surgical management within a multimodality setting, the results of the present study support previous data, showing that sarcoma treatment should be conducted in specialized sarcoma centers^24^.

This study has several limitations, the most important being its retrospective nature. Small sample sizes, especially in subgroup analyses, necessitate cautious interpretation of all significant findings. The long inclusion period presents another limitation, because advances in RPS treatment over the years may have influenced oncological outcomes and may bias the findings of this study. However, bias was reduced as much as possible through stratification by type of surgery as compared with timing of surgery (as in previous studies^16,17^), and by using multivariable analysis to correct for confounders. The interpretation of multivariable regression analysis is limited and validation in larger prospective multicentre cohorts is necessary. Given the rarity and heterogeneity of RPS, retrospective studies have historically served as a key source of comparative data^25^. Single-centre analyses necessitate cautious interpretation of findings, particularly regarding the generalizability of results. Nevertheless, this cohort aligns well with major RPS data sets in terms of tumour characteristics, patient demographics, postoperative morbidity, and recurrence rates, supporting the representativeness of the findings^2,26,27^. Increasing patient numbers in future studies through collaborative efforts, such as those facilitated by the Transatlantic Australasian Retroperitoneal Sarcoma Working Group (TARPSWG), holds considerable potential for clarifying unresolved questions. The growing availability of prospective multinational multicentre data sets, such TARPSWG’s Retroperitoneal Sarcoma Registry (RESAR)^22^, promises to significantly advance future research efforts.

## Supplementary Material

zraf050_Supplementary_Data

## Data Availability

Data available upon reasonable request.
