# Supplementary material for: Histology-driven tailoring of surgical approaches in retroperitoneal soft tissue sarcoma: retrospective cohort study
Source: BJS Open. 2025 May 13;9(3):zraf050. doi: 10.1093/bjsopen/zraf050 (PMC12070264; doi:10.1093/bjsopen/zraf050)
Supplement: zraf050_Supplementary_Data [file zraf050_supplementary_data.docx]

**Title**

Histology-driven tailoring of surgical approaches in retroperitoneal soft-tissue sarcoma: retrospective cohort study

**Authors**

Julian Musa, MD^1,2,3,4*^, Franziska Willis, MD^1,2*^ Ingmar F. Rompen, MD^1^, Julian-C. Harnoss, MD^1^, Thomas G. P. Grünewald, MD, PhD^3,4,5^, Mohammed Al-Saeedi, MD^1^, Markus W. Büchler, MD^1,6^, Martin Schneider, MD^1,2§^

Author affiliations:

1 Department of General, Visceral, and Transplantation Surgery, University Hospital Heidelberg, Heidelberg, Germany

2 Current address: Department of General, Visceral, Thoracic, and Transplantation Surgery, University Hospital Giessen and Marburg, Giessen, Germany

3 Division of Translational Paediatric Sarcoma Research (B410), German Cancer Research Centre (DKFZ), Heidelberg, Germany

4 Hopp-Children’s Cancer Centre (KiTZ), Heidelberg, Germany

5 Institute of Pathology, Heidelberg University Hospital, Heidelberg, Germany

6 Current address: Botton-Champalimaud Pancreatic Cancer Centre, Champalimaud Foundation, Lisbon, Portugal

*Authors share first authorship

**Corresponding author**

Martin Schneider, MD

Department of General, Visceral, Thoracic, and Transplantation Surgery

University Hospital Giessen and Marburg

Rudolf Buchheim Str. 7, 35392 Giessen, Germany

Phone +49-641-985-47001

Email [Martin.Schneider@chiru.med.uni-giessen.de](mailto:Martin.Schneider@chiru.med.uni-giessen.de)

**Supplementary Materials - Index**

| **Supplementary Figures and Tables** |  |
| --- | --- |
| Figure S1 |  |
| Figure S2  Table S1  Table S2  Table S3 |  |
|  |  |

**Supplementary Figures and Tables**

**
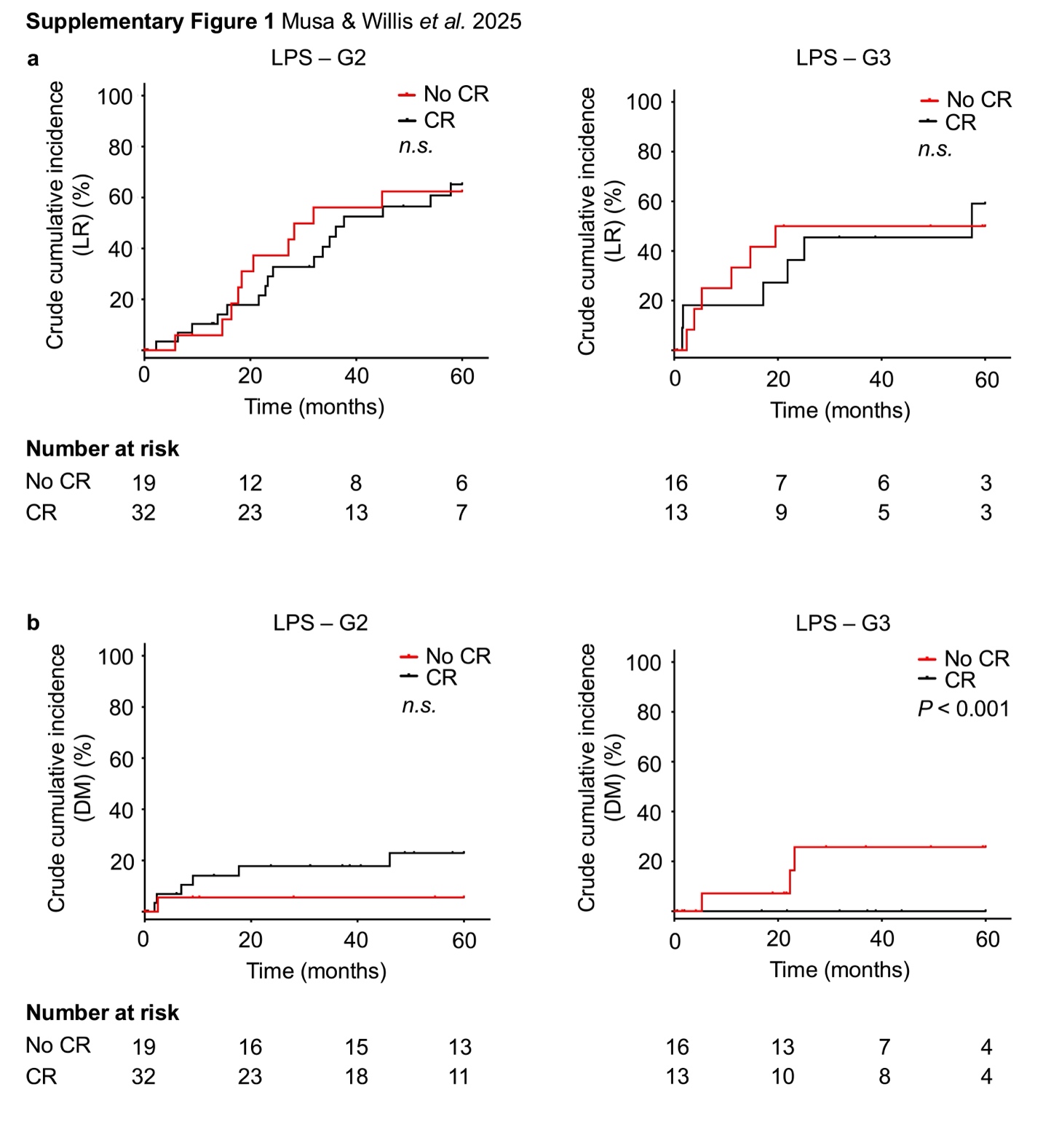
**

**Figure S1: 5-year crude cumulative incidence of local recurrences (LR) (a) and distant metastases (DM) (b) of primary G2 and G3 retroperitoneal liposarcoma (LPS) patients depending on whether comprehensive resection (CR) was performed.** Significance levels given as determined by Fine-Gray test. Analyses separately depicted for G2 LPS (left-hand panels) and G3 LPS (right-hand panels). n.s. = not significant.

**
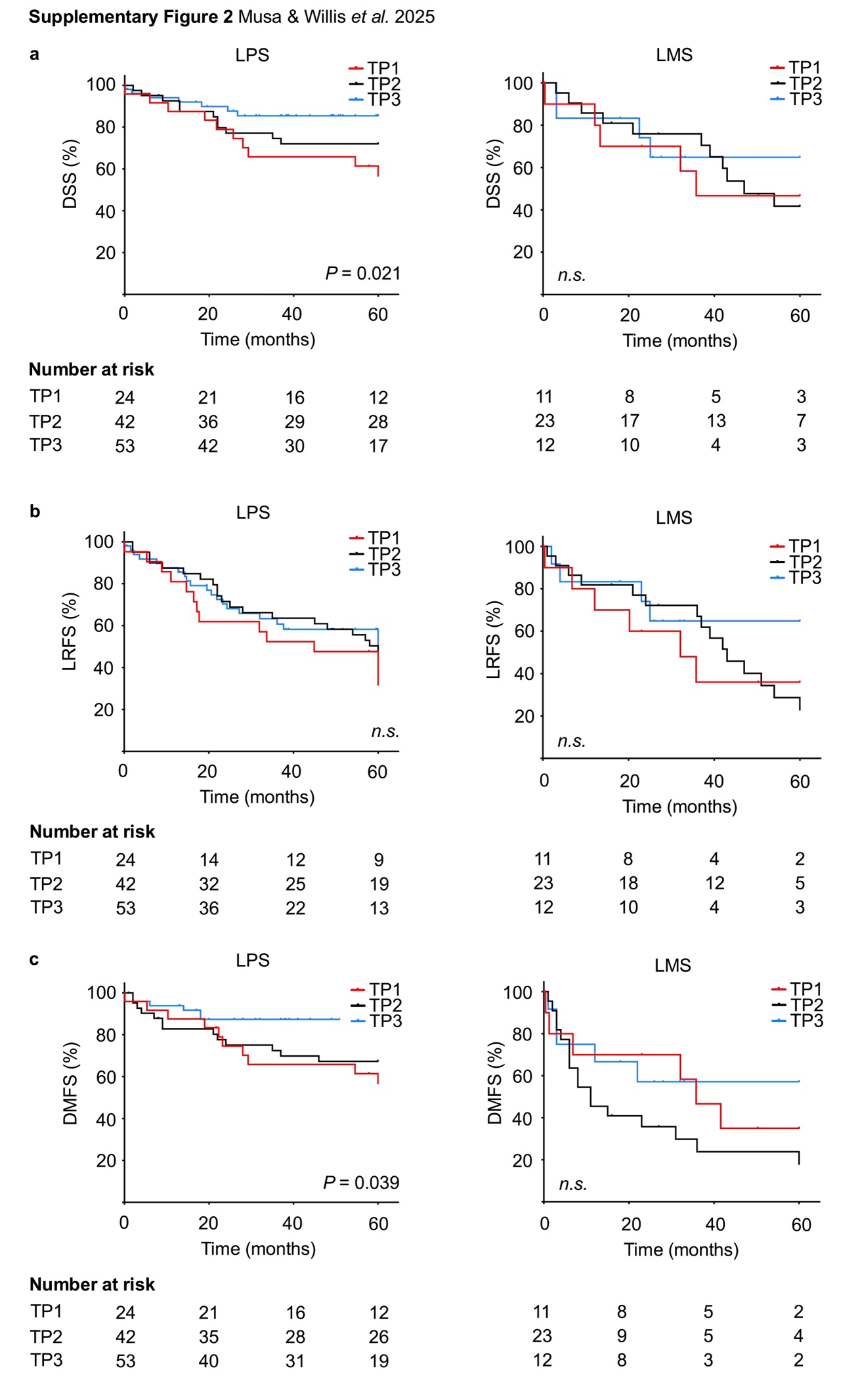
**

**Figure S2: 5-year disease-specific survival (DSS) (a), local recurrence-free survival (LRFS) (b), and distant metastasis-free survival (DMFS) (c) of primary retroperitoneal liposarcoma (LPS) and leiomyosarcoma (LMS) patients depending on the time period (TP) of operation.** Kaplan-Meier analyses separately depicted for LPS (left-hand panels) and LMS (right-hand panels). All significance levels determined by log-rank test. n.s. = not significant.

| perfoRmed COMPREHENSIVE RESECTIONS [%] | 2002 - 2007 | 2008 - 2013 | 2014 - 2019 |
| --- | --- | --- | --- |
| LPS | 20.8 | 52.4 | 71.7 |
| LMS | 18.2 | 47.8 | 75.0 |

**Table S1: Percentages of performed comprehensive resections (CRs) over the time.** Separately shown for primary retroperitoneal liposarcoma (LPS) and leiomyosarcoma (LMS) in the indicated time periods.

| G2-LPS | DSS | | | | LRFS | | | | DMFS | | |
| --- | --- | --- | --- | --- | --- | --- | --- | --- | --- | --- | --- |
|  | **HR** | **95% CI** | ***p*-value** | **HR** | | **95% CI** | ***p*-value** | **HR** | | **95% CI** | ***p*-value** |
| Age (1^st^ Diagnosis) | 1.07 | 1.01-1.13 | 0.019 | 0.99 | | 0.97-1.02 | 0.640 | 1.1 | | 1.00-1.11 | 0.035 |
| Resection status  R0/R1 vs. R2 | 0.09 | 0.01-0.82 | 0.033 |  | |  |  | 0.2 | | 0.03-1.97 | 0.186 |
| COMPREHENSIVE  Resection  yes vs. no |  |  |  | 0.86 | | 0.40-1.80 | 0.667 |  | |  |  |

| G3-LPS | DSS | | | | LRFS | | | | DMFS | | |
| --- | --- | --- | --- | --- | --- | --- | --- | --- | --- | --- | --- |
|  | **HR** | **95% CI** | ***p*-value** | **HR** | | **95% CI** | ***p*-value** | **HR** | | **95% CI** | ***p*-value** |
| Age (1^st^ Diagnosis) | 1.10 | 1-1.2 | 0.045 | 1.11 | | 1.03-1.20 | 0.009 | 1.10 | | 1.00-1.20 | 0.046 |
| Resection status  R0/R1 vs. R2 |  |  |  |  | |  |  |  | |  |  |
| COMPREHENSIVE  Resection  yes vs. no | 0.19 | 0.04-0.93 | 0.040 | 0.51 | | 0.17-1.51 | 0.224 | 0.19 | | 0.04-0.93 | 0.041 |

**Table S2: Multivariable Cox-regression analysis of 5-year disease-specific survival (DSS), local recurrence-free (LRFS), and distant metastasis-free (DMFS) of primary retroperitoneal liposarcoma (LPS) depending on tumour grading.** Data separately depicted for G2 and G3 LPS. *P*-values as determined by multivariable Cox regression analysis. CI = confidence interval, HR = hazard ratio.

| DSS | LPS | | | LMS | | |
| --- | --- | --- | --- | --- | --- | --- |
|  | **HR** | **95% CI** | ***p*-value** | **HR** | **95% CI** | ***p*-value** |
| Age (1^st^ Diagnosis) | 1.07 | 1.02-1.11 | 0.003 |  |  |  |
| Resection status  R0/R1 vs. R2 |  |  |  | 4.64 | 0.52-41.17 | 0.168 |
| TIME period |  |  | 0.054 |  |  |  |
| TP1 vs. TP3 | 3.51 | 1.24-9.92 | 0.018 |  |  |  |
| TP2 vs. TP3 | 2.51 | 0.94-6.68 | 0.065 |  |  |  |
| Tumour grade |  |  | 0.197 |  |  | 0.361 |
| G1 vs. G3 | 0.12 | 0.01-0.94 | 0.044 |  |  |  |
| G2 vs. G3 | 0.62 | 0.27-1.41 | 0.255 | 2.73 | 0.55-13.62 | 0.221 |
| n.a. VS. G3 | 0.55 | 0.14-2.19 | 0.394 | 3.08 | 0.65-14.53 | 0.154 |

| LRFS | Lps | | | lms | | | |
| --- | --- | --- | --- | --- | --- | --- | --- |
|  | **HR** | **95% CI** | ***p*-value** | | **HR** | **95% CI** | ***p*-value** |
| Age (1^st^ Diagnosis) | 1.01 | 0.99-1.04 | 0.290 | | 1.01 | 0.98-1.04 | 0.536 |
| TIME period |  |  | 0.961 | |  |  |  |
| TP1 vs. TP3 | 1.07 | 0.49-2.36 | 0.864 | |  |  |  |
| TP2 vs. TP3 | 1.10 | 0.56-2.14 | 0.783 | |  |  |  |
| Tumour grade |  |  | 0.002 | |  |  | 0.594 |
| G1 vs. G3 | 0.08 | 0.02-0.37 | 0.001 | |  |  |  |
| G2 vs. G3 | 0.87 | 0.46-1.63 | 0.661 | | 1.38 | 0.37-5.13 | 0.632 |
| n.a. VS. G3 | 0.21 | 0.05-0.96 | 0.044 | | 1.87 | 0.52-6.70 | 0.339 |

| DMFS | LPS | | | LMS | | |
| --- | --- | --- | --- | --- | --- | --- |
|  | **HR** | **95% CI** | ***p*-value** | **HR** | **95% CI** | ***p*-value** |
| Age (1^st^ Diagnosis) | 1.06 | 1.02-1.1 | 0.005 | 0.98 | 0.95-1.02 | 0.289 |
| Resection status  R0/R1 vs. R2 |  |  |  |  |  |  |
| TIME period |  |  | 0.050 |  |  | 0.341 |
| TP1 vs. TP3 | 3.21 | 1.16-8.94 | 0.025 | 0.96 | 0.27-3.33 | 0.944 |
| TP2 vs. TP3 | 2.87 | 1.11-7.38 | 0.029 | 1.76 | 0.63-4.92 | 0.283 |
| Tumour grade |  |  | 0.210 |  |  | 0.534 |
| G1 vs. G3 | 0.11 | 0.01-0.9 | 0.040 |  |  |  |
| G2 vs. G3 | 0.78 | 0.35-1.73 | 0.543 | 1.27 | 0.37-4.42 | 0.703 |
| n.a. VS. G3 | 0.57 | 0.14-2.23 | 0.416 | 1.83 | 0.57-5.88 | 0.312 |

**Table S3: Multivariable Cox-regression analysis of 5-year disease-specific survival (DSS), local recurrence-free (LRFS), and distant metastasis-free (DMFS) of primary retroperitoneal liposarcoma (LPS) and leiomyosarcoma (LMS) depending on the time period (TP) of operation.** Data separately depicted for LPS and LMS. *P*-values as determined by multivariable Cox regression analysis. CI = confidence interval, HR = hazard ratio.
